# Genomic and RT-qPCR analysis of trimethoprim-sulfamethoxazole and meropenem resistance in *Burkholderia pseudomallei* clinical isolates

**DOI:** 10.1371/journal.pntd.0008913

**Published:** 2021-02-16

**Authors:** Marine Schnetterle, Olivier Gorgé, Flora Nolent, Aïda Boughammoura, Véronique Sarilar, Cécile Vigier, Sophie Guillier, Lionel Koch, Nicolas Degand, Vincent Ramisse, Xavier Tichadou, Maria Girleanu, Anne-Laure Favier, Eric Valade, Fabrice Biot, Fabienne Neulat-Ripoll

**Affiliations:** 1 Bacteriology Unit, UMR-MD1 INSERM 1261, French Armed Biomedical Research Institut, Brétigny-sur-Orge, France; 2 Ecole du Val de Grace, Paris, France; 3 Molecular Biology Unit, French Armed Biomedical Research Institut, Brétigny-sur-Orge, France; 4 Laboratoire de bactériologie, Hôpital de l’Archet, Centre Hospitalier Universitaire de Nice, Nice, France; 5 DGA MNRBC- Le Bouchet, Division Biologie, ABIO, Vert-le-Petit, France; 6 Imagery Unit, Departement of plateforms and technology research, French Armed Biomedical Research Institut, Brétigny-sur-Orge, France; University of Texas Medical Branch, UNITED STATES

## Abstract

**Background:**

Melioidosis is an endemic disease in southeast Asia and northern Australia caused by the saprophytic bacteria *Burkholderia pseudomallei*, with a high mortality rate. The clinical presentation is multifaceted, with symptoms ranging from acute septicemia to multiple chronic abscesses. Here, we report a chronic case of melioidosis in a patient who lived in Malaysia in the 70s and was suspected of contracting tuberculosis. Approximately 40 years later, in 2014, he was diagnosed with pauci-symptomatic melioidosis during a routine examination. Four strains were isolated from a single sample. They showed divergent morphotypes and divergent antibiotic susceptibility, with some strains showing resistance to trimethoprim-sulfamethoxazole and fluoroquinolones. In 2016, clinical samples were still positive for *B*. *pseudomallei*, and only one type of strain, showing atypical resistance to meropenem, was isolated.

**Principal findings:**

We performed whole genome sequencing and RT-qPCR analysis on the strains isolated during this study to gain further insights into their differences. We thus identified two types of resistance mechanisms in these clinical strains. The first one was an adaptive and transient mechanism that disappeared during the course of laboratory sub-cultures; the second was a mutation in the efflux pump regulator *amrR*, associated with the overexpression of the related transporter.

**Conclusion:**

The development of such mechanisms may have a clinical impact on antibiotic treatment. Indeed, their transient nature could lead to an undiagnosed resistance. Efflux overexpression due to mutation leads to an important multiple resistance, reducing the effectiveness of antibiotics during treatment.

## Author summary

*B*. *pseudomallei* is a Gram-negative bacterium that causes melioidosis, a tropical disease. The mortality rate is high, the treatment long and harsh, and the therapeutic arsenal is limited due to the natural resistance of the bacteria to antibiotics. Eleven percent of melioidosis cases are chronic. Here, we studied a chronic melioidosis case in a French male patient who lived in Malaysia in the 70s. *B*. *pseudomallei* was identified in 2014 and in a relapse in 2016. Analysis revealed several strains from the same clinical sample with different morphotypes and divergent antibiotic-resistance profiles. Two atypical multidrug resistance profiles were observed for two strains: one possessed multiple resistance to trimethoprim-sulfamethoxazole, fluoroquinolones, and chloramphenicol and the other multiple resistance to fluoroquinolones and meropenem. Trimethoprim-sulfamethoxazole or meropenem resistance have rarely been described in clinical cases and are probably underdiagnosed. Here, we show that trimethoprim-sulfamethoxazole resistance can be transient in clinical strains and easily lost in the laboratory after sub-culture during identification, resulting in an underestimation of trimethoprim-sulfamethoxazole resistance and therapeutic failure. We also identified a mutation in the AmrAB-OprA efflux pump regulator, leading to high level meropenem resistance, but this resistance is also transient.

## Introduction

*Burkholderia pseudomallei* is the causal agent of melioidosis, which is endemic in southeast Asia and northern Australia. However, recent studies have shown that the endemic area may extend to Southern Asia, Central America, and Middle East [1]. The mortality rate is up to 50%, depending on the endemic area [2]. In 2015, a modeling study estimated that there were 165,000 cases of human melioidosis with 89,000 estimated deaths [1]. Melioidosis is a multifaceted disease, with symptoms ranging from acute septicemia to multiple chronic abscesses.

*B*. *pseudomallei* is known for its intrinsic resistance to many antibiotics, such as macrolides, several β-lactams, polymyxins, and aminoglycosides. Such resistance considerably reduces the therapeutic arsenal [3]. The treatment of melioidosis is aggressive and composed of two phases: acute and eradication. During the acute phase, parenteral ceftazidime or a carbapenem are given for at least 14 days. The eradication phase consists of oral treatment with trimethoprim/sulfamethoxazole for a recommended 20 weeks [4]. Most strains remain susceptible to trimethoprim-sulfamethoxazole, ceftazidime, and amoxicillin-clavulanic acid [5–7] and only few studies have reported decreased susceptibility to carbapenems [8, 9]. All known resistance mechanisms in *B*. *pseudomallei* are chromosomally encoded. Resistance to ceftazidime is often due to the loss of PBP-3 [10] or mutations in the β-lactamase *penA* gene (*BPSS0946)* [11–15]. More recently, duplication of *penA* was described in ceftazidime resistance in chronic infections [16], and in a strain isolated from a Thai patient [17]. Meropenem susceptibility can be altered by the loss of the regulation of RND efflux pumps, especially the AmrAB-OprA pump [8]. Finally, trimethoprim-sulfamethoxazole resistance is often observed in association with BpeEF-OprC overexpression or mutations in the folate pathway [18,19]. Resistant strains should not be neglected, especially given that the relapse risk rate of recurrent melioidosis is 25% [20]. Acquired resistance during melioidosis treatment is rare and the mechanisms are poorly understood and may be underestimated [21].

One of the main mechanisms of the multidrug resistance (MDR) phenotype of *B*. *pseudomallei* is antibiotic efflux by RND efflux pumps. There are approximately 10 putative RND pumps in the genome of *B*. *pseudomallei* [22,23], but only three have been described: AmrAB-OprA [24], BpeAB-OprB [25,26], and BpeEF-OprC [27]. Mutations in efflux pumps or their regulators have often been reported and may explain the MDR phenotype [8,16,18,28]. Bacteria may also adapt by modulating the expression of their efflux pumps after low-level antibiotic exposure [29]. In such cases, multiple resistance is unstable in antibiotic-free medium. This mechanism of adaptive resistance was described by Fernandez *et al*. as “an ability of a bacterium to survive an antibiotic insult due to alteration in gene in/or protein expression as a result to an environmental trigger, e.g., stress, nutrient conditions, growth state, and subinhibitory levels of the antibiotics themselves” [30].

Here we report the case of a 96-year-old male patient with chronic carriage of melioidosis. The 1^st^ respiratory sample from this patient dates from 2014 and allowed the growth of four isolates with divergent morphotypes and antibiotic susceptibilities. Two isolates (named A1 and A2) were characterized by large smooth colonies, and two others isolates (named B1 and B2) by small rough colonies. Antibiogram analysis showed B1 to be the most resistant isolate, with resistances to: quinolones, chloramphenicol, and trimethoprim-sulfamethoxazole. In 2016, a new respiratory sample from this patient was still positive for *B*. *pseudomallei*. We did not observe divergence of morphotypes nor antibiograms and this unique isolate (named C) showed atypical resistance to meropenem. Microscopic observation was carried out to investigate a possible correlation between macroscopic observations, resistance and the cell membrane of the strains.

We investigated how the divergence in antibiotic susceptibility may have occurred in these related isolates. We hypothesized that: (*i*) the isolates came from the same ancestral strain, *(ii)* the divergence in antibiotic susceptibility was due to the involvement of the BpeEF-OprC efflux pump for the trimethoprim-sulfamethoxazole-resistant isolate B1, *(iii)* the meropenem resistance for C strain is due to a mutation in the *amrR* gene. More generally, we wanted to conduct a descriptive study of the mechanisms that may be at the origin of the difference in resistance for these isolates. We thus performed comparative whole genome sequencing (WGS) and RTq-PCR analysis of the genes encoding several efflux-pumps. WGS analysis confirmed the close genetic relationship between these isolates. No mutations in any efflux pump genes were identified that could explain the marked multiple resistance observed in the B1 isolate and we observed no efflux pump overexpression by RTq-PCR. The mechanisms behind such divergence are still unidentified but we observed the resistance of isolate B1 to be transient and lost after several passages in antibiotic-free medium. This suggests that *Burkholderia pseudomallei* harbors adaptive resistance, which must be considered in the clinic. We also determined that atypical meropenem resistance in the C isolate was due to a mutation in the *amrR* gene, a regulator of the AmrAB-OprA efflux pump, leading to marked overexpression of the pump. Surprisingly we have observed a decrease meropenem resistance after 10 sub-cultures for this strain, but still within MIC resistance limits.

## Methods

### Ethics statement

The samples are the property of the Acting National Reference Center for Melioidosis in France. We received the approval of our Institutional Review Board (LDR Bukholderia pathogènes), and anonymized the samples. The *Burkholderia pseudomallei* K96243 strain has been used as reference [19].

### Strains and bacterial growth

Clinical strains were grown on trypticase soy (TS) agar medium, in broth (BD Difco Laboratories), or on Ashdown’s agar medium at 37°C in biosafety level 3 (BSL3) laboratory (Table 1). The morphological analysis was performed from Ashdown-medium cultures because the observed divergence was more striking on this medium.

**Table 1:** *B*. *pseudomallei* strains used.

Strain	Isolated from	Year of isolation	ST	Reference
**A1**	Bronchoalveolar lavage	2014	414	this study
**A2**	Bronchoalveolar lavage	2014	414	this study
**B1**	Bronchoalveolar lavage	2014	414	this study
**B2**	Bronchoalveolar lavage	2014	414	this study
**C**	Bronchoalveolar lavage	2016	414	this study
**K96243**	Unknown	1996	10	[19]

### Antibiotic susceptibility testing

The minimal inhibitory concentrations (MICs) of all the antibiotics tested were determined using the Etest procedure (bioMérieux) on Mueller-Hinton II agar plates (BD Difco Laboratories). We used a 0.5 McFarland inoculum according to the manufacturer’s recommendation. Results were obtained after incubation at 37°C for 24 h and are expressed as mg/L.

### Sub-culturing

One sub-culture step consisted of harvesting 2 to 3 colonies from a TS Agar medium plate, with a 10 μl inoculation loop, inoculating 5 ml TS liquid medium free of antibiotics, and incubating the inoculum for 6 h at 37°C with 200 rpm agitation. Then, a new TSA plate without antibiotics was inoculated using a 10 μl inoculation loop soaked in the TS liquid medium culture. New colonies were used to perform a new sub-culture step and antibiograms.

### DNA isolation and sequencing

Bacteria were grown overnight in 3 mL TS broth. DNA isolation was performed using the DNeasy Blood and tissue kit (Qiagen) according to the manufacturer’s instruction. DNA concentration and purity were determined by spectrophotometric analysis (NanoDrop 1000 spectrophotometer, Thermo Fisher Scientific). DNA integrity was determined by agarose gel electrophoresis.

Paired-end 150 bp x 2 libraries were prepared using the NEB Next Ultra DNA library Prep Kit for Illumina (New England Biolabs) and sequenced on an Illumina Nextseq 500 sequencer (Illumina).

### Genomic analyses

Read quality was checked with FastQC software (V00.11.4) [31]. Read quality-based trimming was performed using Cutadapt (V1.3.1) with the following parameters: -q 20 -m 30 [32]. SPAdes assembler (V3.10.1) [33] was used to perform the *de novo* assemblies and Quast (V4.5) [34] for the quality control, alignment and visualization of the *de novo* assembly. Trimmed reads were mapped on the K96243 genome using BWA (V0.7.15) [35] and SAMtools (V0.1.19) [36]. The resulting.bam files and.gff files were visualized using Geneious software (R11, BioMatters).

The trimmed reads were directly analyzed by the DiscoSNP++ (V2.2) [37] program and Bionumerics 7.6.3, with default parameters to determine the single nucleotide polymorphisms (SNPs) and small insertions/deletions (indels) between isolates. The genome of *B*. *pseudomallei* K96243 (assembly number: GCF_000011545.1 accession number NC_006350.1 and NC_006351.1) was used as a reference for sequencing and annotation. The SnpEFF [38] program was used to predict the impact of SNPs and indels on the protein sequences.

Sequence typing was determined *in silico* by multi-locus sequence typing (MLST) [39, 40]. We used whole genome data to attribute the sequence type (ST) for each strain using SRST2 V0.2.0 [41] with the MLST database for *B*. *pseudomallei* [42]. Sequence typing was confirmed by Sanger sequencing (Genewiz).

The strains have been deposited in the NCBI BioProject database under accession number no. PRJNA526444.

### RNA isolation

Bacteria were grown in 5 mL TS broth until mid-exponential phase (OD600nm = 0.6 ± 0.1). All extractions were performed six times for each strain and each condition.

A 2 mL aliquot of each culture was pelleted for 1 min at 10,000 rpm at room temperature. The bacterial pellet was resuspended in 1 mL of RNAprotect Bacteria Reagent (Qiagen). Total RNA was isolated using the RNeasy lipid and tissue mini-kit (Qiagen) according to the manufacturer’s instructions and genomic DNA eliminated by a 15 min incubation with RNase-free DNase I set (Qiagen) during the isolation procedure. RNA was stored at -80°C, RNA concentration and purity were determined by spectrophotometric analysis (NanoDrop 1000 spectrophotometer, Thermo Fisher). RNA integrity was determined using a Bioanalyzer RNA 6000 Nano kit (Agilent).

### Reverse transcription and real-time PCR

Reverse transcription was performed with the reverse transcription core kit (Eurogentec). cDNAs were synthesized using the same volume of total RNA to minimize RT-qPCR variability due to differences between samples [43]. RNA extracts < 600 ng were supplemented with yeast tRNA (Ambion) to obtain a final concentration of 600 ng. The RT reaction was performed in a final volume of 30 μL. Samples were incubated at 25°C for 10 min and then at 48°C for 30 min. The reverse transcriptase was inactivated at 95°C for 5 min and the samples transferred to ice and incubated for at least 5 min. Samples were then diluted two-fold with water and stored at -80°C.

PCR primers were designed using “Primer3 plus” with the reference sequence of *B*. *pseudomallei* K96243 [44] (Table 2). All primers used in this study were designed on the K96243 *Burkholderia pseudomallei* reference genome. The efficiency of all primers was tested by blasting their sequences against those of the genomes of the clinical strains that were sequenced and analyzed.

**Table 2:** Primers and optimized PCR conditions used for transcriptional analysis.

Primer	sequence 5’-> 3’	quantity unit [a]	annealing (°C)	annealing (s)	[MgCl2] (mM)	[primer] (μM)
**RpsL_F1**	TCGTACATCGGCGGTGAAG	15U	54	7	5	0,4
**RpsL_R1**	CCGCGAACCATGTGGTAAC					
**RpoD_F2**	TGCTGCAGGAAGGCAACCTCG	15U	55	5	4	0,4
**RpoD_R2**	AAATCGCCTGACGAATCCACC					
**RimM_F2**	CGACAACGGCGTGCATTCGATC	15U	50	7	4	0,4
**RimM_R2**	GCCTTCACGTACACGCCGACGAAC					
**DnaK_F2**	CGAAATCAACCTGCCGTACATC	15U	50	7	5	0,4
**DnaK_R2**	CGGGTGATCTTCAGATTCAAGTG					
**RumA_F2**	CATCGTCGCGGTCGGCCACA	15U	58	5	4	0,4
**RumA_R2**	AGCGCAGTTCGGGCTTCACTTC					
**AmrB_F1**	TCGATCAACGTGCTGACGATG	15U	53	9	5	0,6
**AmrB_R1**	GCAGCTTCTCCTCGACCATCAG					
**BpeB_F1**	CTCGTCGCGTTGATTCTGAC	15U	53	9	5	0,4
**BpeB_R1**	AGTTGAAGGTGCGGTTGAAC					
**BpeF_F2**	GGCTTCAACAAGGTGTTCCATC	30U	58	5	4	0,5
**BpeF_R2**	GGAGATACAGGCCGAGCATCACG					

[a]: 30 U: 1μL of cDNA in 4 μL water; 15 U: 0.5 μL of cDNA in 4.5 μL water.

Optimal conditions were determined on a pool of all cDNA samples. Primer sequences and optimal PCR conditions are shown in Table 2. qPCR was carried out using the Lightcyler Fast Start Sybr Green kit (Roche) in a final volume of 20 μL in a LightCycler 2.0 apparatus (Roche). Cqs were calculated using LightCycler software V4.1 (Roche).

Among the five candidate reference genes (*rpoD*, *rpsL*, *dnaK*, *rimM*, and *rumA*) only the three most stable (*rimM*, *rpsL* and *rumA*) were selected for normalization using Genorm [45]. Standardization and quantification were performed relatively to the geometric means of these three reference genes as described by Willems *et al*. [46].

All statistical analyses were performed using GraphPad Prism (GraphPad Software, Inc.). At least four biological replicates were used for statistical analyses based on the Kruskal Wallis multiple comparison test and Dunn’s test. We considered p-values below 0.05 to be significant.

### Transmission electron microscopy analyses

Bacteria were grown in 5 ml TS at 37°C and 200 rpm until mid-exponential phase (OD600nm = 0.6 ± 0.1). Bacteria (2 ml) were pelleted by centrifugation for 2 min at 3,000 rpm and inactivated with glutaraldehyde (Sigma)[47]. Samples were fixed with 2.5% (v/v) glutaraldehyde (EMS) in cacodylate buffer (0.1 M, pH 7.4, 2% sucrose, with CaCl_2_ and MgCl_2_ (Merck Millipore) overnight at 4°C. After washing, samples were post fixed with 1% (v/v) osmium tetroxide in cacodylate buffer for 1 h at room temperature under a chemical hood and covered with aluminum foil. Then, samples were stained for 1 h at 4°C with 2% (v/v) uranyl acetate and further dehydrated progressively higher concentrations of ethanol. Samples were then embedded in Epon LX112 (Inland Europe) resin and subjected to polymerization at 60°C for 48 h. Ultrathin sections (100 nm) were cut using a Reichert Ultracut S ultramicrotome (Leica Microsystems) and placed onto 300 mesh copper grids. Sections were then double stained with 2% uranyl acetate and lead citrate (EMS). High-resolution transmission electron microscopy was performed with a CM10 microscope operating at 100 kV and equipped with a CCD Erlanghsen 1000 Gatan camera. No filtering procedure was applied to the images.

## Results

### Clinical data about the patient with a chronic melioidosis

In 2014, a 96-year-old male patient attended the pneumology ward of the University Hospital of Nice, France, for low-abundance hemoptysis. His clinical history included chronic obstructive pulmonary disease and bronchiectasis after tuberculosis in the 1970s while living in Malaysia. Microbiological analysis of sputum and bronchoalveolar lavage retrieved 1 x 10^6^ CFU/mL and 1 x 10^3^ CFU/mL of *Burkholderia pseudomallei* respectively, allowing the diagnosis of melioidosis. Bacterial identification was performed using MicroFlex LT (Bruker Daltonics, Bremen, Germany) and the SR *database* containing *security-relevant* bacteria. The patient received 1 gram of amoxicillin-clavulanic three times daily for 10 days *per os*. In 2016, the patient had a pulmonary exacerbation due to *Haemophilus influenzae*, successfully treated with 1 gram of amoxicillin-clavulanic acid, three times daily for 10 days in January, September and December. However, *B*. *pseudomallei* was still identified in a clinical sample. Chronic colonization with *B*. *pseudomallei* was diagnosed due to positivity of cultures of all respiratory specimens collected from this patient (2014 and 2016, Fig 1).

**Fig 1:**
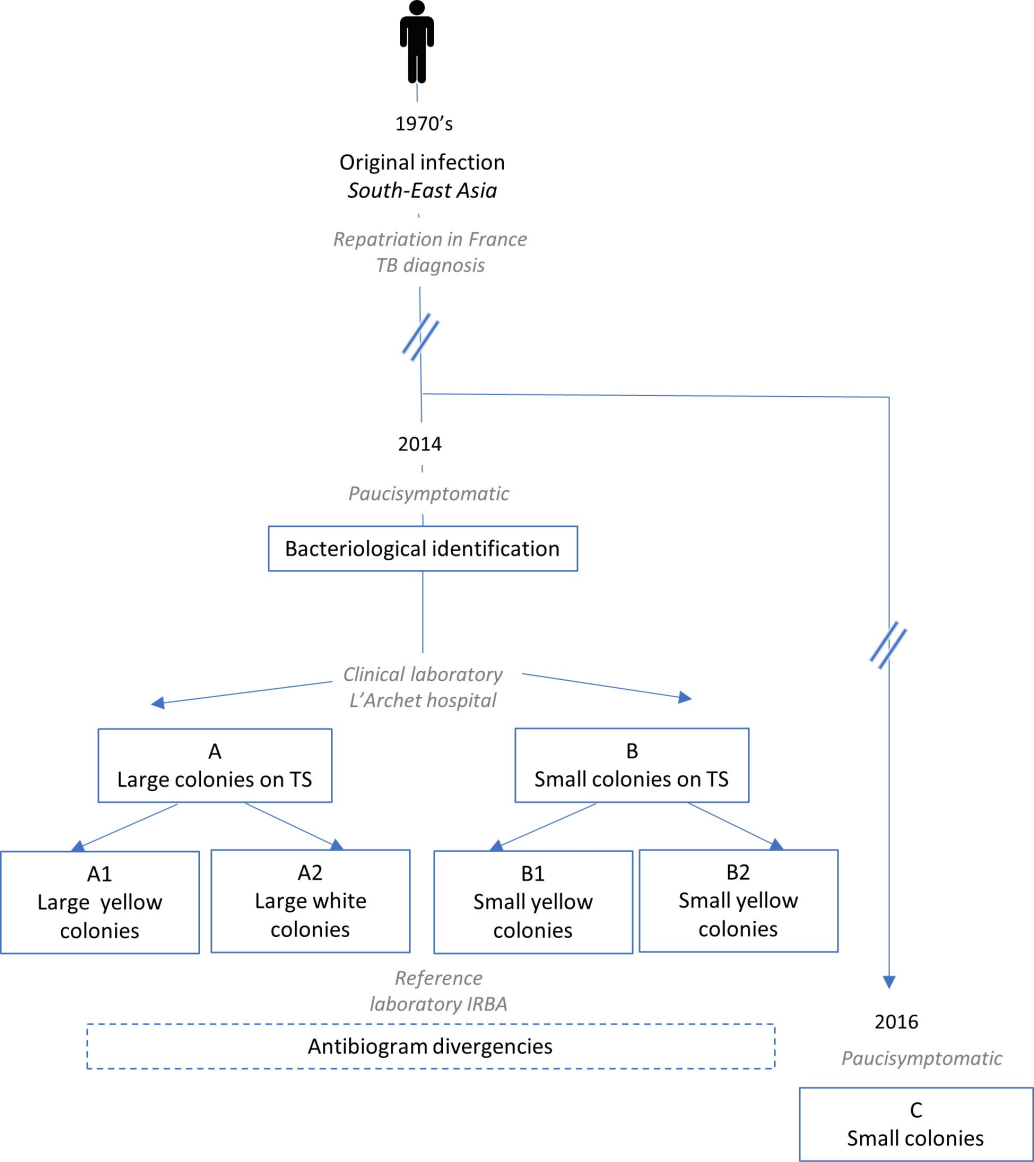
Schematic representation of the clinical cases and studied strains. In 2014, in a single sample, four strains of *Burkholderia pseudomallei* were isolated from a paucisympotomatic old man, and differenced by their morphology and pigmentation (A1, A2, B1, B2). In 2016, a new strain was isolated (C).

### Observed phenotypic differences allowed for variant differentiation

**Fig 2:**
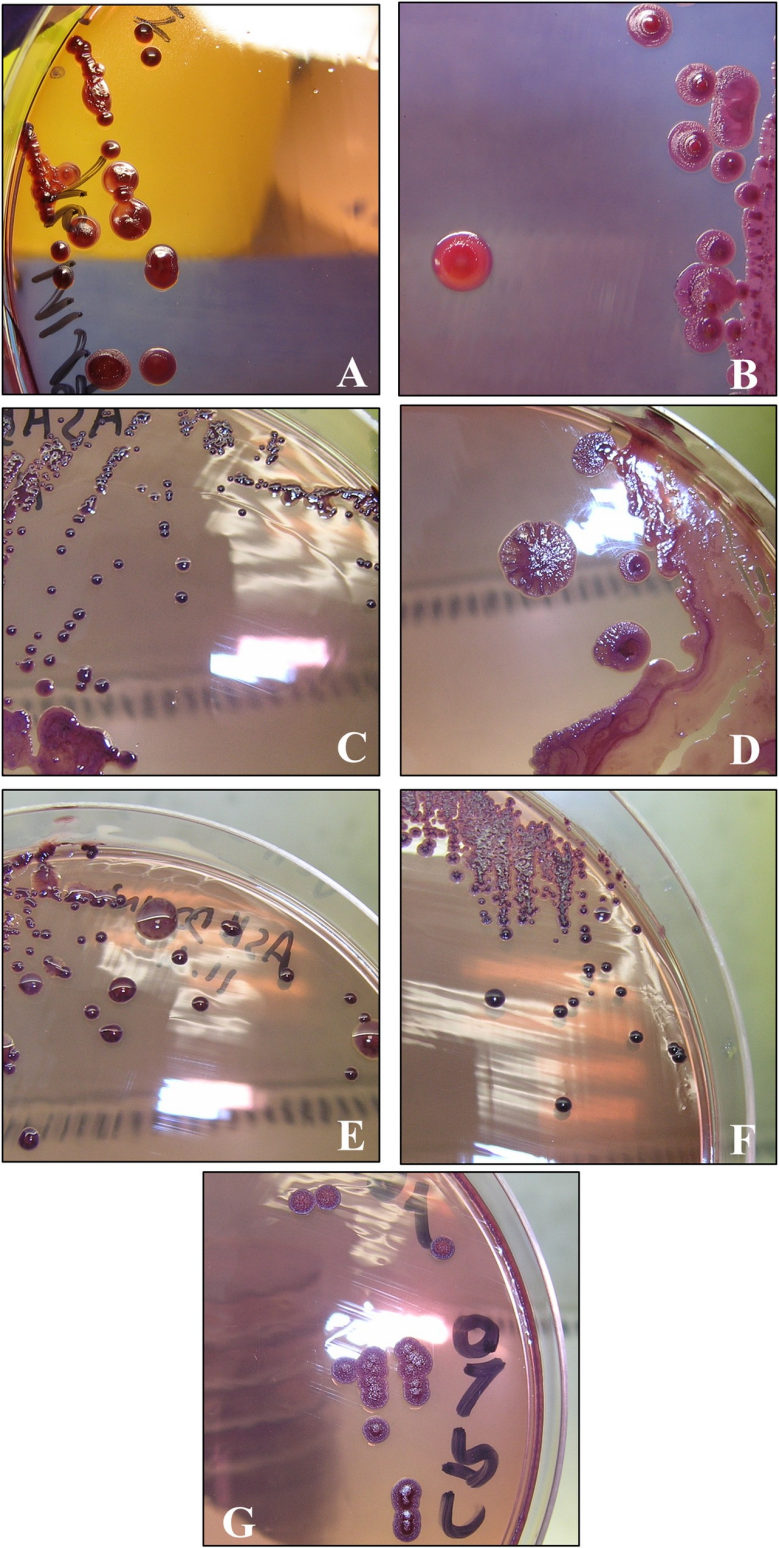
Morphotype of *B*. *pseudomallei* clinical strains after five days of culture on Ashdown’s agar medium at 37°C. A: A1 strain; B: A2 strain; C: B1 strain; D: B1 strain sub-cultured five times (B1R5); E: B2 strain; F: C strain; G: C strain sub-cultured 10 times (CR10).

Two morphotype variants were isolated from the fibroscopy sample at the first admission of this patient in 2014: one was characterized by large smooth colonies (A) and the other by small colonies (B). Morphological divergences were observed and the two isolates separated into four variants: A1 and A2 were differentiated by their pigmentation on TS medium, and B1 and B2 had different antibiogram patterns (Fig 2 and Table 3). For diagnostic confirmation at the French Armed Forces Biomedical Research Institute (IRBA), we used bacterial culture in Ashdown medium for specific identification, as described by Ashdown in 1979 [48] (Fig 2). Two years later a new sample was sent to IRBA. This sample contained only one morphotype, consisting of very small colonies (named strain C) (Figs 1 and 2).

**Table 3:** Antibiotic susceptibility of the *B*. *pseudomallei* strains. The MIC were determined by Etest procedure (bioMerieux) and are expressed in mg/L.

strains	MIC (mg/L)													
	β-lactams				quinolones				aminoglycosides		sulfamides		phenicol	cycline
	AMX	AMC	CAZ	MEM	CIP	LVX	NAL	NOR	GEN	AMK	SXT	TMP	CHL	DOX
**A1**	32	1	1	0.25	3	4	32	64	24	256	1	>32	24	2
**A2**	32	1	0.75	0.38	4	4	32	48	16	96	1.5	>32	16	1.5
**B1**	>256	1.5	1.5	1.5	>32	>32	>256	>256	96	>256	>32	>32	256	4
**B1R5**	256	1	0.5	1	2	4	24	48	16	96	1	>32	12	4
**B2**	>256	1.5	2	1	16	24	256	128	64	>256	1.5	>32	96	4
**C**	>256	1.5	1.5	>32	>32	>32	192	>256	>1024	>256	1.5	>32	24	8
**CR5**	>256	1.5	1	32	16	32	192	256	>1024	>256	1	>32	12	6
**CR10**	64	1.5	0.75	4	4	32	192	192	256	>256	1.5	>32	8	2

Antibiotic abbreviations: AMX: amoxicillin; AMC: amoxicillin-acid clavulanic; CAZ: ceftazidime; MEM: meropenem; CIP: ciprofloxacin; LVX: levofloxacin; NAL: nalidixic acid; NOR: norfloxacin; GEN: gentamicin; AMK: amikacin; STX: trimethoprim-sulfamethoxazole; TMP: trimethoprim; CHL: chloramphenicol; DOX: doxycycline

### Differences observed in strains susceptibility allowed the identification of uncommon trimethoprim-sulfamethoxazole (cotrimoxazole) and meropenem resistance

Antibiograms permitted us to distinguish four variants (Table 3). The A strains were less resistant, A2 being the least resistant, whereas the B strains showed higher Minimal Inhibitory Concentrations (MICs) for quinolones (ciprofloxacin, levofloxacin, nalidixic acid, and norfloxacin), aminoglycosides (gentamicin and amikacin), and amoxicillin. B1 was the most resistant strain showing multiple resistance against quinolones, trimethoprim-sulfamethoxazole, and chloramphenicol. An antibiogram was also performed on the C variant, which had very high MICs for quinolones, similar to those of strains B1 and B2 (Table 3). However, strain C was more susceptible to chloramphenicol and trimethoprim-sulfamethoxazole than strain B1, and more resistant to gentamicin and meropenem.

### MLST analysis

MLST analysis revealed that all strains analyzed in this study belong to ST414, suggesting that they are closely genetically related, despite differences in morphotype and antibiotic susceptibility. This sequence type has already been observed in Malaysian strains thus correlating with the endemic region where this patient had lived [49].

### Microscopic observations

**Fig 3:**
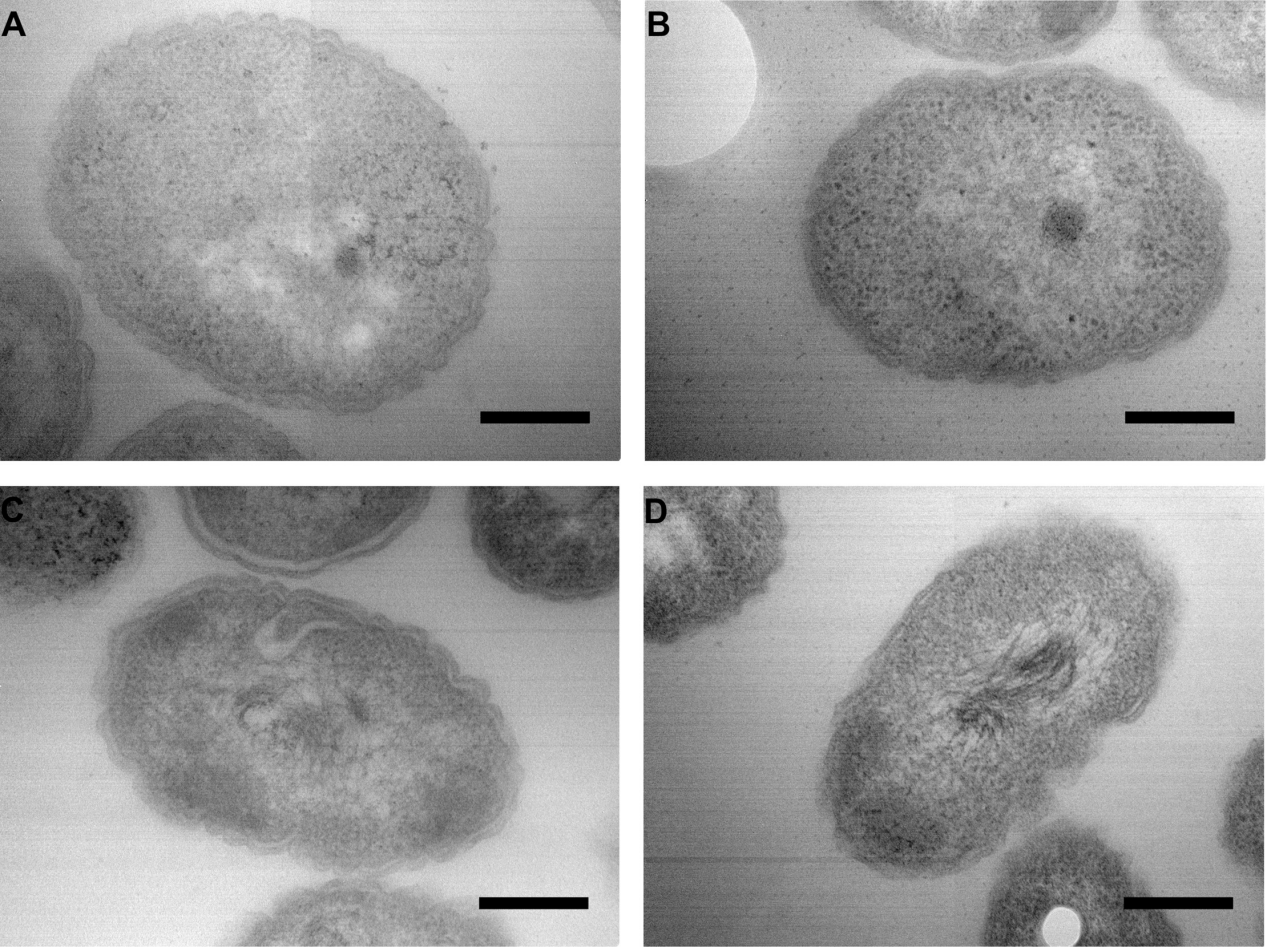
Microscopic observations by TEM (transmission electron microscopy). Observation of the three strains isolated in 2014 and 2016 (**A**: strain A2; **B**: strain B1; **C**: strain C), and reference strain (**D**: strain K96243). The scale bar represents 250 nm. The observations presented here are without trimethoprim induction, the same pictures were obtained with trimethoprim induction.

The different morphotypes and levels of antibiotic resistance that we observed prompted us to investigate whether there was a relationship between them, and the possible involvement of the cell-wall structure of the strains. We thus examined the strains by transmission electron microscopy (TEM). There were no differences in appearance of the cell walls between the strains, whether they are more or less resistant to antibiotics. The Fig 3 represent the microscopy observation of the four strains.

### Genomic differences between strains analyzed by WGS

We performed a comparative genomic analysis to determine whether genetic differences could explain the phenotypic and antibiotic differences. We used two approaches: a whole genome comparison at the structural level, and an analysis of Single Nucleotide Polymorphism (SNP) and insertion/deletion (indel) analysis.

Whole genome analysis allowed us to identify a large deletion on chromosome 2 of approximatively 210 Kbp in the A1, A2, and B2 strains. This deletion is localized between nucleotides 2061202 and 2273364 in the K96243 reference genome and flanked by two regions containing the same eight-nucleotide pattern: CGGGCCGC. This deletion may have occurred by recombination at this site.

**Fig 4:**
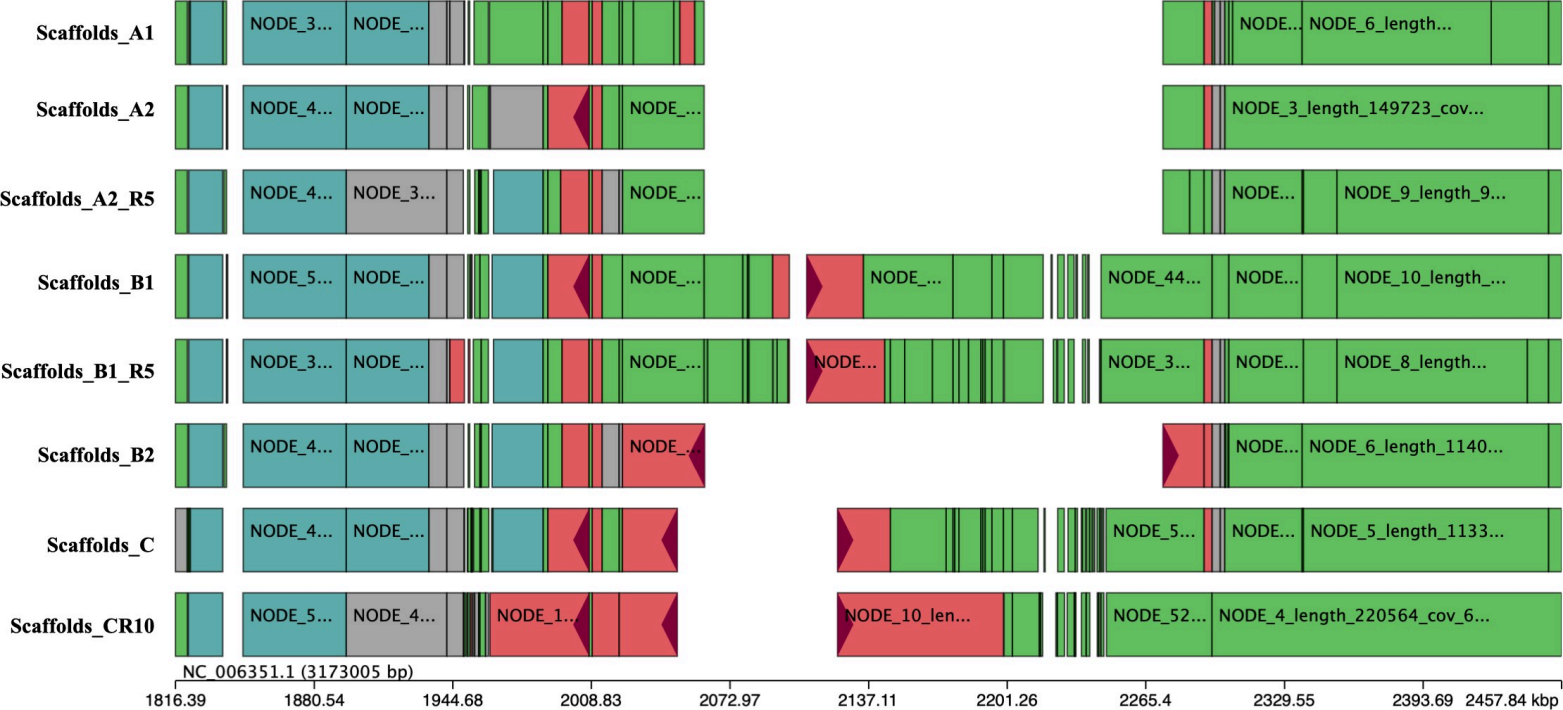
Representation of the deleted region in clinical strains. The strains scaffolds were aligned against the K96243 reference genome. There is a focus of the deletion region, K926243 chromosome 2 is represented by a black line and notated NC_006351.1.

This deleted chromosomal region contains 145 genes (loci between BPSS1511 and BPSS1654 on K96243). Most of these genes encoded in this region are involved in virulence: T3SS clusters 2 (BPSS1610-BPSS1629) and 3 (BPSS1516-BPSS1554), seven global regulator genes: four *lysR* (BPSS1643; BPSS1640; BPSS1586 and BPSS1559), one *marR* (BPSS1556), and two *araC* (BPSS1520 and BPSS1610); and four putative “two-component systems” composed of predicted sensor kinase and response regulator. Two-component systems are involved in the regulation of the other genes present in the neighborhood, and implicated in virulence [50]. The remaining genes encoded in the region are involved in bacterial metabolism. This region is partially present in strains C and CR10, localized between nucleotides 2,048,593 and 2,122,665, on chromosome 2 (Fig 4). It covers the loci BPSS1502 to BPSS1563, containing T3SS3, part of T6SS1 (T6SS1 BPSS1496-BPSS1551), *folE (*GTP cyclohydrolase of the folate pathway*)*, and *marR* (transcriptional regulator).

**Fig 5:**
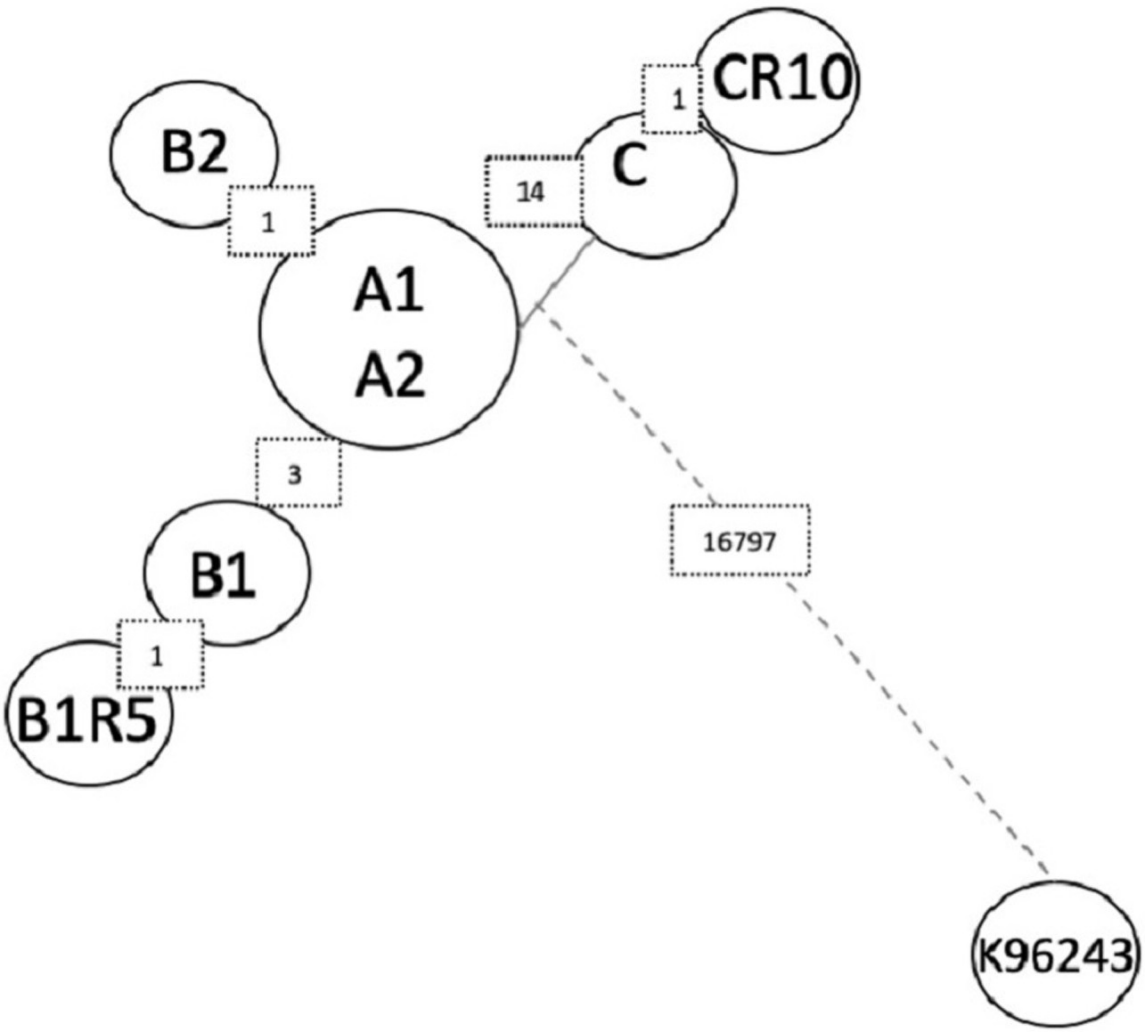
Dendrogram representation of SNPs found in all clinical isolates of this study and compared to the K96243 reference strain, obtained using Bionumerics software. The numbers on the squares indicate the number of SNPs between each isolate and strain.

We tested the clonality of these strains by performing a *de novo* SNP analysis and small indel detection using DiscoSNP++ software. The SNPs are all listed in Table 4 (SNPs determined in the coding regions by DiscoSNP++) and are represented in a dendrogram which was created using Bionumerics software (Figs 4 and 5). There are no SNPs between A1 and A2 genomes. The B1 genome has three specific SNPs relative to the A strains (in loci BPSL1363, BPSL1560 and BPSL2409) and possesses one SNP in common with strain C, which is located in the BPSL2325 gene, encoding an N-acetylglutamate synthase. Strain B2 possesses two specific SNPs, one at the same locus (BPSL2325) but at another position and the second in an intergenic region at position 3,293,345 on chromosome 1. Strain C differs from the other strains by 12 SNPs and three indels in the coding regions. Only one SNP is located in an efflux pump regulator, a common mechanism of antibiotic resistance. The C strain *amrR*, regulator of the AmrAB-OprA efflux pump, has a mutation at position 533, consisting of a CC insertion. This insertion leads to a frameshift, which delocalizes the stop codon 243 bp further and replaces the last 45 amino acids with 127 other ones. Strain C also differs by three SNPs in intergenic regions at positions 772,505 and 1,047,324 on chromosome 1 and 2,514,699 on chromosome 2.

**Table 4:** SNPs determined in the coding regions by DiscoSNP and Bionumerics software.

	LOCALISATION	GENE	GENE PRODUCT	MUTATIONS							MUTATION EFFECT
				A1	A2	B1	B1R5	B2	C	CR10	
Chromosome 1	577094	**BPLS0525**	putative protein	A	A	A	A	A	1520 A>G	1520 A>G	Leu507Pro
	772505	772505	-	T	T	T	T	T	T>C	T>C	-
	1047324	1047324	-	C	C	C	C	C	C>A	C>A	-
	1592084	**BPSL1363**	phosphate transport system like protein	C	C	285 C>T	285 C>T	C	C	C	Leu95Leu
	1811140	**BPSL1560**	hypothetical protein	G	G	179 G>T	179 G>T	G	G	G	Ala60Glu
	1967418	**BPSL1687**	putative protein	G	G	G	G	G	493 G>C	493 G>C	Gly165Arg
	2055472	**BPSL1743**	ArcA	A	A	A	A	A	146 A>C	146 A>C	Asn49Thr
	2095298	**BPSL1775**	Iron uptake receptor	C	C	C	C	C	2216 C>T	2216 C>T	Met739Ile
	2096152	**BPSL1775**		C	C	C	C	C	1363 G>A	1363 G>A	Gly455Arg
	2097391	**BPSL1775**		G	G	G	G	G	124 C>T	124 C>T	His42Tyr
	2097460	**BPSL1775**		C	C	C	C	C	55 C>T	55 C>T	
	2199533	**BPSL1846**	putative protein	G	G	G	G	G	139 G>A	139 G>A	Arg47Cys
	2313254	2313254	-	G	G	G	G	G	G	G>C	
	2811582	**BPSL2325**	N-acetylclutamate synthase	G	G	G	G	1094 G>A	G	G	Cys365Tyr
	2912606	**BPSL2409**	ABC Transporter ATP-Binding protein	T	T	854 T>C	854 T>C	T	T	T	Leu285Pro
	3337083	**BPSL2789**	WcbR	G	G	G	G	G	35G>A	35G>A	Ser12Phe
	3827935	**BPSL3226**	NusG	T	T	T	T	T	50 T>C	50 T>C	His17Arg
Chromosome 2	1210864	**BPSS0916**	N-hydroxyarylamine O-acetyltransferase	G	G	G	G	G	764G>C	764G>C	Gly255Ala
	2354508	**BPSS1715**	GltA	C	C	C	C	C	1219C>T	1219C>T	Asp407Asn
	2698543	2698543	-	G>A	G>A	G>A	G>A	G>A	G	G	-
				**INSERTION & DELETION**							
	LOCALISATION	GENE	GENE PRODUCT	A1	A2	B1	B1R5	B2	C		MUTATION EFFECT
Chromosome 1	2152840	**BPLS1805**	AmrR						533_534dupCC	533_534dupCC	frameshift
	1967552	**BPSL1687**	Hypothetical protein	582_596 TGGCTGCGCTGGTGA	582_596 TGGCTGCGCTGGTGA	582_596 TGGCTGCGCTGGTGA	582_596 TGGCTGCGCTGGTGA	582_596 TGGCTGCGCTGGTGA			Gly195_Pro199del
	2811321	**BPSL2325**	N-acetylglutamate synthase			843_844delGC	843_844delGC		843_844delGC	843_844delGC	frameshift
Chromosome 2		**BPSS0369**	bacterioferritin ferredoxin protein						206_delT	206_delT	frameshift

### Efflux-pump expression analyses

Multiple resistance observed in the B1 strain suggests that a nonspecific mechanism may be involved. We sought to confirm this hypothesis by performing RT-qPCR analysis to test for potential differences in efflux pump expression between our strains. We analyzed the relative expression of the three main RND transporters of *B*. *pseudomallei*: *amrB*, *bpeB*, and *bpeF*. We have chosen to perform these analyses for the three strains: A2 the less resistant strain, B1 the most resistant, and C with the atypical meropenem resistance.

**Fig 6:**
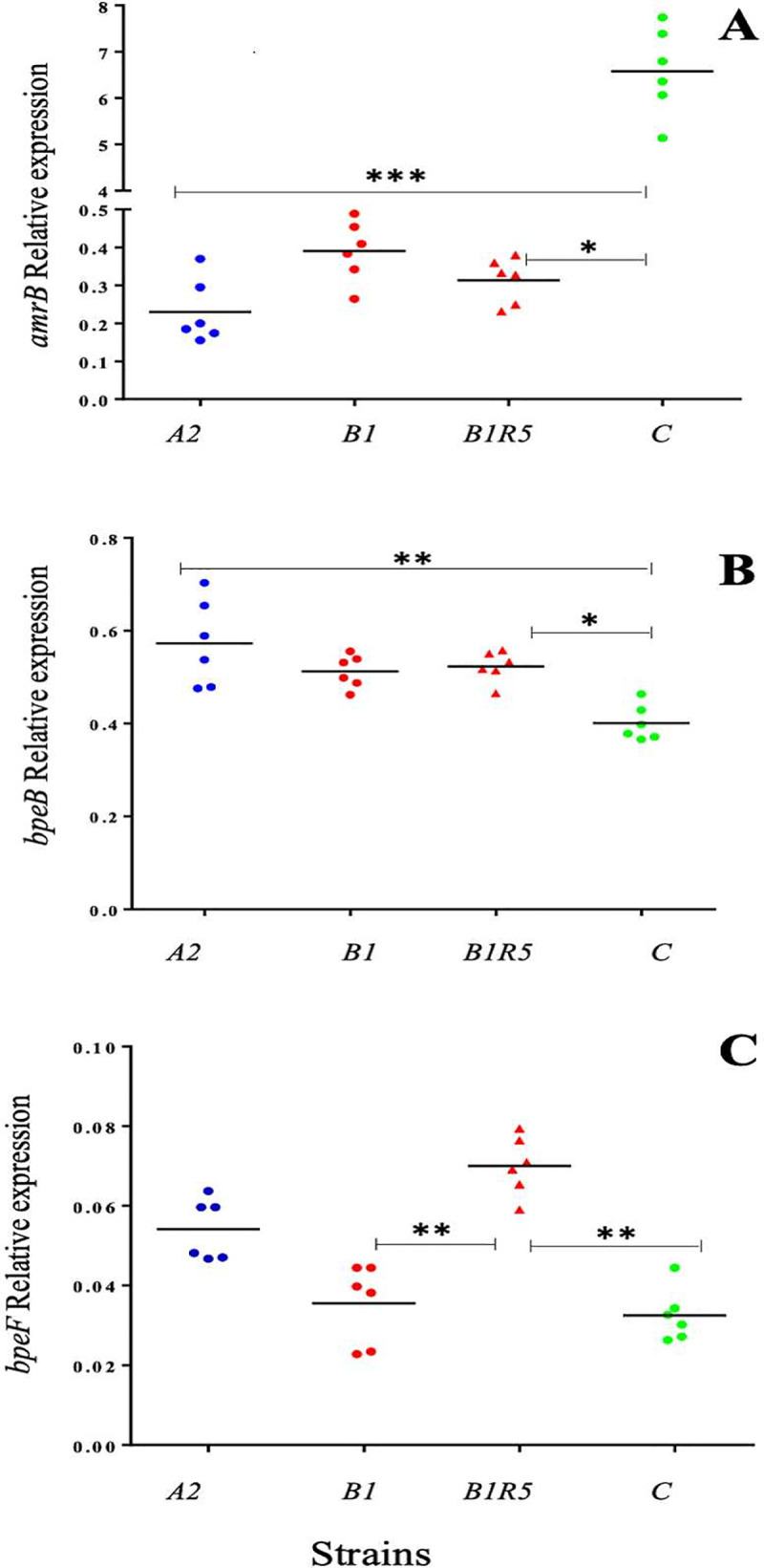
Relative expression of RND efflux-pump transporters in *B*. *pseudomallei* clinical strains. Relative expression of *amrB* (**A**) *bpeB* (**B**), *bpeF* (**C**). Bars represent the median. Statistical analysis was performed using the Kruskal Wallis test for multiple comparisons. Statistical two-group comparisons were performed using Dunn’s test and are represented on the graphs with bars and *.

The *AmrB* transporter was highly overexpressed in strain C (Fig 6), approximately 29-fold higher than in strain A2 and 17-fold higher than in strain B1. Statistical differences are observed for *bpeB* with a low expression in the C strain, and an overexpressed *bpeF* for B1 strain. There was no statistical difference between strains A2 and B1 for any of the three transporters analyzed.

### Stability of the resistance in B1 cotrimoxazole resistant strain and C meropenem resistant strain

The mechanism behind the atypical resistance in B1 was still unclear after WGS analysis and RTq-PCR. We thus hypothesized that such resistance could be easily lost without antibiotic pressure.

We analyzed the stability of the resistance of the most resistant strain, B1, by several sub-cultures in antibiotic-free medium as describe previously. After 5 rounds of sub-culture, MIC of the derivative variant, named B1R5, for quinolones, chloramphenicol, and trimethoprim-sulfamethoxazole decreased markedly (Table 3). This result suggests that the multiple resistance observed in B1 strain in 2014 was unstable, and moreover the morphotype changed on Ashdown’s medium, becoming very similar to A strains morphotype, with large smooth colonies (Fig 2D). Then, we performed the same sub-culturing experiment on strain C to observe also a potential reversible resistance in this strain. Antibiotic resistance after five rounds of sub-culture (variant named CR5) was unchanged, but the MICs for meropenem and gentamicin decreased after 10 rounds of sub-culture (variant named CR10) (Table 3). Morphotype analysis of strain C showed a shift towards larger rough colonies (Fig 2G), similar to the phenomenon observed for B1R5.

Gene duplication and amplification (GDA) is a mechanism sometimes involved in reversible resistance [51] and responsible of acquired ceftazidime resistance in *B*. *pseudomallei* [17]. We thus sought if B1 isolate reversible resistance was due to this GDA mechanism by bioinformatics analysis with Bionumerics software. However, our analysis did not allow us to observe the presence of GDA for any of the three efflux pumps genes analyzed here.

WGS showed that B1R5 differed from B1 in a non-coding region of chromosome 1: position 3824689, 135 nucleotides upstream of BPSL3321 (DNA directed RNA polymerase beta chain (Table 4, Fig 4). CR10 compared to the parental strain has just one SNP in a non-coding region of chromosome 1 at position 2313254, an intergenic region 11 nucleotides upstream of the *pheS* gene (BPSL1941). This gene is a Phenylalanine-tRNA ligase alpha subunit involved in protein biosynthesis, and have no implication in resistance mechanism.

Without explanations about these reversible resistance by GDA and WGS, we analyzed the expression of the *amrB*, *bpeB*, and *bpeF* genes in strain B1R5. We have observed an increase of *bpeF* expression in the range of 1.97-fold-higher than the parental strain B1.

## Discussion

Here, we report a chronic case of melioidosis in a male patient who lived in an endemic region for several years in the 70’s and was repatriated in France during this period after being diagnosed with tuberculosis, and he never went back in this region. Chronic melioidosis is uncommon, representing only 11% of melioidosis cases [2]. This patient was first diagnosed for melioidosis in 2014 after a routine examination. A second examination in 2016 revealed that he was still positive for *B*. *pseudomallei*. We do not know when this patient was infected with *B*. *pseudomallei*, nor whether it was misdiagnosed as tuberculosis in the 70’s, as melioidosis is known to mimic tuberculosis. All strains isolated during this study belong to ST414, a sequence type previously found in Malaysia, Singapore, and Thailand [49].

All five variants isolated from this patient exhibited different morphologies and antibiograms patterns. Polymorphic cultures arising from clinical samples are not rare in *B*. *pseudomallei* infections, and seven different *B*. *pseudomallei* morphotypes have been described [52,53]. Morphotypes changes can appear under antibiotic pressure, and this phenomenon has been described with ciprofloxacin and ceftazidime at sub-inhibitory concentrations [52]. Moreover, antibiotic exposure can provoke morphological changes like filamentation [54]. This morphological change is reversible without antibiotic pressure, except for an initial ofloxacin induction. A filamentation induction by ceftazidime leads to small colony variant formation which are known to have high minimum inhibitory concentration level [55,56]. Our strains presented different morphotypes and antibiotics divergencies, thus, we examined the variants A2 and B1 the two most different, variant C isolated, and the *B*. *pseudomallei* reference strain K96243, by transmission electron microscopy to observe a possible correlation between cell-wall structure, morphotype, and level of resistance. We did not observe any microscopic differences between the strains that could explain their macroscopic morphotypes and levels of resistance (Fig 3).

Whole genome SNP analysis revealed several genomic differences between the variants. Only the two A variants did not differ from each other. Very few SNP were detected between the clinical strains: three SNPs between the strains isolated in 2014, and 14 SNPs with this group and the C strain isolated in 2016. In comparison there is 16,797 SPNs between these clinical strains and the K96243 reference strain. These findings, as well as the ST identity, suggest that the variants came from the same ancestral strain. Indeed, the patient never returned to the endemic area after leaving in the 70’s, raising questions about the pathogen micro-evolution in the host during the intervening years [16,57].

We identified a multiple resistance profile with high MICs for trimethoprim-sulfamethoxazole, chloramphenicol, and quinolones in the B1 variant. Such a profile is often associated with efflux-pump overexpression, particularly BpeEF-OprC [28,29,58,59]. This suggests that differences in the antibiotic resistance between the isolated variants were probably due to differences in efflux-pump expression, which can give rise to acquired resistance, when due to mutation, or adaptive resistance [30].

We performed WGS of the variants isolated in 2014 to determine whether the trimethoprim-sulfamethoxazole, quinolone, and chloramphenicol multiple resistance observed in strain B1 was due to a mutation located in efflux-pump genes, or there regulators [19,60]. Strain B1 differed by five SNPs and B2 by one from the non-MDR A1 and A2 variants. Strain B1, which was more resistant than strain B2 against trimethoprim-sulfamethoxazole, differed from strain B2 by six SNPs (Table 4). None were located in efflux pumps known to be involved in antibiotic resistance in *B*. *pseudomallei*. Sequencing also showed that the less resistant variants, A1, A2, and B2, possess a 210 kb deletion on chromosome 2. This region contains T3SS2 and T3SS3, which are crucial bacterial virulence factors [61], and the BsaN regulator of T3SS3. This regulator is also involved in the regulation of other virulence factors and T6SS1 [62]. This deletion contains several LysR and one MarR family regulator, a *folE* gene (BPSS1514), and one quorum-sensing system (BPSS1569-BPSS1570). However, the deleted region in these variants does not contain efflux-pump genes.

Furthermore, RT-qPCR analysis comparing strain B1 to the A2 non-MDR variant showed that the BpeEF-OprC is not overexpressed in strain B1 (Fig 6). Thus, we compared the expression of the two others main RND transporters of *B*. *pseudomallei*, *amrB* and *bpeB*, to determine whether a pump other than BpeEF-OprC could be involved. We did not observe overexpression of either of these transporters in strain B1.

The multiple resistance to trimethoprim-sulfamethoxazole, quinolones, and chloramphenicol in strain B1 was unstable and lost after five rounds of sub-culture (B1R5) (Table 3). It is likely that strain B1 exhibits adaptive resistance, which has been described by Fernandez *et al*. as a transient form of resistance due to the alteration of gene or protein expression, resulting from an environmental trigger [30]. Efflux can be altered by transcriptional regulation in bacteria in cases of adaptive resistance [30,63]. This could explain why we were unable to observe the expected BpeEF-OprC overexpression, as BpeEF-OprC overexpression could have been lost under our culture conditions. As frequently described in the literature, trimethoprim-sulfamethoxazole resistance is due to the up-regulation of *bpeF*. Even if results are statistically significant, we did not expect a slightly overexpression of *bpeF* in B1R5 strain relative to B strain. We cannot affirm that the resistance observed for our strains depends only on the 3 efflux pumps studied here.

Variant C was isolated two years after the variants A1, A2, B1 and B2, and is characterized by high quinolones and meropenem resistance and higher MICs for aminoglycosides than those observed for the other variants. WGS showed a SNP in *amrR*, consisting of an insertion at position 533–534, leading to a disruptive frameshift. This mutation could impair the *amrR* repressor and lead to the overexpression of AmrAB-OprA. RTq-PCR showed that the efflux pump is highly overexpressed in variant C compared to variants A2 and B1 (Fig 6). Overexpression of AmrAB-OprA could explain the atypical meropenem resistance of strain C and the high MICs for aminoglycosides. Indeed, the involvement of AmrAB-OprA overexpression in a meropenem resistant strain was recently described by Sarovich *et al*. [8]. We were surprised for the very high MIC observed for meropenem antibiotic in comparison with Sarovich *et al*., then we repeated eight MIC measurements in 2017 and 2019 with different batches of Etest and MH-II medium, and we always observed the same results. Strain C also has a 74-kb deletion in chromosome 2, which shares a region in common with the deletion present in the strains A and B2. The T3SS3 system is also deleted in strain C. In addition, we observed a deletion of T6SS1, which is essential for virulence in a murine model [64,65]. Strain C also has a deletion of the *folE* gene (BPSS1514), as do the strains A and B2, and it is also less resistant to trimethoprim-sulfamethoxazole than strain B1. A modification of the folate pathway is a possible cause for this type of resistance, *folE* and *folM* genes are in an operon and the deletion of *folE* could impair *folM* expression. *folM* mutation has been observed in a trimethoprim resistant strain [18]. As the *folE* gene is found at other loci in the *B*. *pseudomallei* genome: BPSS0040 and BPSS1248 [23], we cannot state that FolE is involved here in this resistance, especially in a transient resistance.

Here, we have shown that trimethoprim-sulfamethoxazole resistance can be transient in a clinical strain (B1) and spontaneously lost in the laboratory by sub-culturing. This suggests that the number of trimethoprim-sulfamethoxazole-resistant strains in the clinic may have been underestimated because of the instability of their antibiotic resistance. Eradication therapy with oral trimethoprim-sulfamethoxazole is recommended for at least 20 weeks to reduce the risk of relapse. The failure to identify resistant strains during diagnosis is problematic and prolonged exposure to this antibiotic could lead to the selection of a stably resistant strain. It is possible that transient resistance is the first step towards stable resistance due to mutations acquired during continuous exposure to antibiotic pressure. As observed in this study, transiently resistant strains appear to have a divergent morphology as microcolonies. This suggests that assessment of colony variants by the clinician microbiologists after the isolation of *B*. *pseudomallei* from the patient should be reinforced. Two years after the first identification of A2 and B1 strains, we identified in a new sample, a meropenem-resistant variant (C), which one showed overexpression of the AmrAB-OprA efflux pump due to a mutation in the *amrR* repressor. Meropenem is one of the antibiotics used during the acute phase of melioidosis treatment, and such resistance could explain some therapeutic failures. It is however surprising that this meropenem resistance, despite the presence of *AmrR* mutation, is transient after 10 sub-cultures.

This study raises various questions because we did not observe the same resistance mechanisms generally described in the literature. Indeed, the analysis of DNA sequences obtained do not allow us to understand the mechanisms of transient resistance, we do not observe any mutations in regulators (apart *AmrR* for C strain), nor any compensatory or back mutations. Then, we focused on efflux pumps regulation, but again, our results are not in agreement with the literature. Our study had not shown an increase of BpeEF-OprC efflux pump expression for the multi-resistant strains. Others mechanisms than efflux pumps could be also associated with its resistance and not detected here. Further transcriptional analyses by total RNA sequencing will be necessary to evaluate the involvement of other putative efflux pumps, other regulators of the *B*. *pseudomallei* genome in antibiotic resistance; and may allow us to identify the genes that are up or down regulated in these strains.

Another point that will have to be investigated is epigenetic. When DNA or RNA could not explain resistance mechanisms, it is obvious that epigenetic mechanisms may contribute to resistance development and its transient character [66], or small regulatory RNAs.

Another question is about the micro-evolution of *B*. *pseudomallei* in patient. We do not know if the C strain is an evolution of the strains isolated in 2014, with a development of meropenem resistance, or if it was present in 2014 but not differentiated by the clinician.

Melioidosis is considered to be a neglected tropical disease and clinicians are often untrained and unaccustomed to identifying *Burkholderia pseudomallei*. In general, antibiograms to measure antibiotic susceptibility are performed on a single colony after identification of *B*. *pseudomallei* by Ashdown culture. Our study shows that is particularly important *i)* to be attentive to the different morphological aspects of colonies and, as far as possible, to test the antibiotic resistance of each morphotype and *ii)* to avoid multiple rounds of sub-culturing before antibiogram analysis, as the resistance may be transient. Non-identified adaptive transient resistance to trimethoprim-sulfamethoxazole could partially explain the differences that are observed between the high rate of relapse and the low rate of the emergence of resistance during treatment.
